# Influence of Soldiers on Exploratory Foraging Behavior in the Formosan Subterranean Termite, *Coptotermes formosanus* (Blattodea: Rhinotermitidae)

**DOI:** 10.3390/insects14020198

**Published:** 2023-02-16

**Authors:** Joseph McCarthy, Arjun Khadka, Hasim Hakanoglu, Qian Sun

**Affiliations:** Department of Entomology, Louisiana State University Agricultural Center, Baton Rouge, LA 70803, USA

**Keywords:** eusociality, division of labor, tunneling behavior, soldier–worker interaction

## Abstract

**Simple Summary:**

Termites are eusocial insects that live in large colonies made up of queens, kings, workers, and soldiers. Queens and kings start new colonies and continually reproduce. Workers are responsible for many crucial roles, including colony husbandry, foraging, and nest construction. Soldiers are adapted for colony defense, usually with large heads and mandibles to ward off predators, but their defensive adaptations prevent them from caring for themselves or performing tasks within the nest. The soldiers of some termite species participate in foraging, either directly by scouting food sources and recruiting workers, or indirectly by influencing worker foraging behavior through their presence. Colonies of the Formosan subterranean termite maintain relatively large soldier proportions compared to termites in their invasive range, but the potential soldier influence on foraging workers has not yet been studied. Since the soldiers of other termite species can influence food exploration, we hypothesized that soldier presence also influences foraging behavior in this species. We compared the exploratory behavior of foraging groups of 100 workers and either 0, 2, 10, or 30 soldiers to determine whether soldier concentration influenced tunnel complexity, tunnel speed, food location, or food collection. In the context of this study, soldier presence did not influence worker foraging behavior, which suggests that workers of the Formosan subterranean termite can maintain foraging efficiency regardless of fluctuations in soldier presence.

**Abstract:**

Termites are eusocial insects that live in organized colonies consisting of reproductives, workers, and soldiers. Soldiers are specialized for defense but are expensive to maintain, as they are incapable of husbandry and must be fed and groomed by workers. The soldiers of several species influence foraging behavior by acting as scouts that initiate foraging or by mediating worker behavioral plasticity during food exploration. These behaviors imply that soldiers may play a keystone role in termite colony function, apart from defense. Subterranean termite workers tunnel through soil in search of food while accompanied by varying proportions of soldiers, depending on the species and colony conditions. Previous studies have shown that soldiers accelerate worker exploratory tunneling behavior in two *Reticulitermes* species, the colonies of which contain fewer than 2% soldiers. This effect, however, is unknown in other subterranean species with different soldier proportions. In this study, we examined the influence of soldiers on exploratory foraging behavior in the Formosan subterranean termite, *Coptotermes formosanus* Shiraki, which is an economically devastating invasive species that maintains a relatively high soldier proportion (about 10%). When 100 foraging workers were grouped with 0, 2, 10, or 30 soldiers in two-dimensional foraging arenas, we found no significant effect of soldiers on the tunnel length, branch pattern, food source interception, or food collected within 96 h. These results suggest that *C. formosanus* colonies maintain food exploration efficiency regardless of soldier proportion variation.

## 1. Introduction

Colonies of subterranean termites (family Rhinotermitidae) nest underground and tunnel through soil in search of cellulose-containing food. Colonies consist of workers, soldiers, and reproductives (the queen and king), and individuals from each caste perform specific roles to ensure colony success [1]. The queen and king are responsible for colony foundation and, once a colony is established, they are cared for while they continually reproduce. Workers fill many important roles, which include nest construction, food collection, nestmate care, and hygienic activity within the nest. Subterranean termite workers can also differentiate permanently into soldiers as needed, and the main role of the soldier caste is colony defense [2]. Soldiers use a variety of defense mechanisms, including chemical defense, physical attack, and phragmotic head capsules, to protect the colony [2,3,4]. The worker–soldier transition involves a tradeoff in the colony, as the soldiers bear modified mandibles that prevent them from performing any of the tasks they fulfilled as workers. Their adaptations also render them unable to feed or groom themselves, so they fully rely on the workers to care for them [2,5]. Because of the energy investment, colonies regulate their soldier proportions within species-specific ranges for optimal colony function [6], and soldier concentrations can deviate based on seasonality, colony age, and a variety of social contexts [7,8,9]. 

Subterranean termite colonies search for and collect food through collective behaviors performed by workers and mediated by both social and environmental conditions, such as colony size, temperature, and food availability [10,11,12,13,14,15]. Foraging typically takes place underground and begins with the exploration of an area. Above-ground foraging is less common and is performed by creating mud tubes that protect the termites from biotic and abiotic stressors. In soil, workers commence the exploratory phase of foraging by tunneling outward from an existing food source or nest chamber. They construct tunnels by excavating soil and depositing the particles elsewhere [16], and the geometry of the tunnel network is optimized for food encounter rate [17]. Once a new food source is found, the next phase involves colonization of the food item. Using trail pheromones laid inside the tunnels, more termites are recruited to aid in food collection [18]. During this process, the collected food items are consumed and transport by workers, who also return to the nest to provision the reproductives and brood [19,20]. 

Foraging is a risky task, as termites must leave the safety of their nest to explore new areas. Termites have a variety of predators and competitors, especially ants and other termite species [3,21,22]. Interactions with predators (especially ants and other opportunistic feeders of termites) and competitors (other termite colonies or wood-nesting ant species) are more likely outside the main nest, and in some cases, these interactions may be fatal for a group of foragers [5]. While soldiers are incapable of food collection or unassisted consumption, they typically accompany workers during foraging activities, and it has been suggested that soldiers play a keystone role in colony function beyond direct colony defense [23,24,25]. Little is understood, however, about the potential keystone role soldiers play among termite species, particularly subterranean species with cryptic foraging behavior. 

Soldiers of some higher termite species (family Termitidae) directly participate in the exploratory phase of foraging. Soldiers of the arboreal termite *Nasutitermes corniger* (Motschulsky) act as scouts by actively searching new areas for food [26]. Once a food source is found, the soldiers lay trail pheromones to recruit more soldiers, and eventually, workers, to collect and transport the food. The soldiers self-regulate their presence while actively protecting the foraging workers as they establish foraging trails and begin collecting food [26,27,28]. Similarly, the subterranean termite *Heterotermes tenuis* (Hagen) has dimorphic soldiers, and the minor soldiers act as scouts that initiate food exploration and direct workers to food sources when foraging above ground [29]. Foraging behavior initiated by minor soldiers was also reported in *Coptotermes intermedius* Silvestri [30]. Soldiers of other subterranean species, however, influence foraging by regulating the behavior of workers. In *Reticultermes hageni* (Banks), soldiers participated in the exploratory phase of foraging by accelerating the initialization of tunneling by workers, and in *R. flavipes* (Kollar), soldier presence increased the tunneling speed of workers and the number of tunnel branches they constructed [25]. Extracts of soldier cuticular hydrocarbons were sufficient to affect tunnel speed in *R. flavipes*, indicating that the behavioral plasticity of workers was influenced by the recognition of solders [25]. In these species, the direct or indirect participation of soldiers in foraging is considered an adaptive strategy to reduce predation risk during food exploration, highlighting the keystone role of the soldier caste [25,26,29].

The Formosan subterranean termite, *Coptotermes formosanus* Shiraki, is an economically devastating structural pest endemic to southeast Asia [31], and is recognized as one of the 100 worst invasive species in the world [32]. The below-ground tunnel networks of a single colony can reach over 100 m [33]. *C. formosanus* are among the subterranean species with a relatively large proportion of soldiers in their colonies, maintaining a soldier concentration of around 10%, though soldier proportion varies depending on the social and environmental conditions [7,8,9,34]. Su & La Fage showed that the proportions of *C. formosanus* soldiers were slightly higher in foraging groups, representing 14–46% of foragers, when compared to those in the nest, representing 7–23% [35]. *C. formosanus* soldiers utilize mechanical defense via mandibular attacks and chemical defense through the secretion of a white sticky fluid from their fontanelle (i.e., frontal gland opening) [36]. 

As a defensive caste, soldiers are energetically costly to maintain and are usually limited to a small proportion of the colony in social insects [2]. The high presence of soldiers in *C. formosanus* may suggest additional roles to colony defense, similar to soldier–worker interactions that mediate food exploration in the subterranean genus *Reticulitermes* and the congeneric *C. intermedius* [25,29]. We hypothesized that *C. formosanus* soldiers would also influence the exploratory phase of foraging. To test this hypothesis, we examined collective foraging behavior during the exploratory phase, considering factors including tunnel length, the number of tunnel intersections (a proxy of branching pattern), the interception of food sources, and total food collection, with groups of 100 workers and either 0, 2, 10, or 20 soldiers.

## 2. Materials and Methods

### 2.1. Termites

Four *C. formosanus* colonies were collected from Bretchel Park in New Orleans, Louisiana (29°54′29″ N, 90°00′32″ W), using milk-crate traps filled with a lattice of 2″ × 2″ kiln-dried pine wood [37]. Foraging groups that consisted of workers and soldiers were collected and used for the experiments within three months of collection. Prior to the experiments, the colonies were maintained in complete darkness at 25 ± 1 °C in clear acrylic containers (38.48 × 45.72 × 22.86 cm^3^) (Pioneer Plastics, North Dixon, KY, USA) with 2 cm of organic soil (Miracle-Gro All Purpose for In-Ground Use, Scotts Miracle-Gro, Marysville, OH, USA) and moistened pine wood blocks.

### 2.2. Foraging Arena Setup

**Figure 1:**
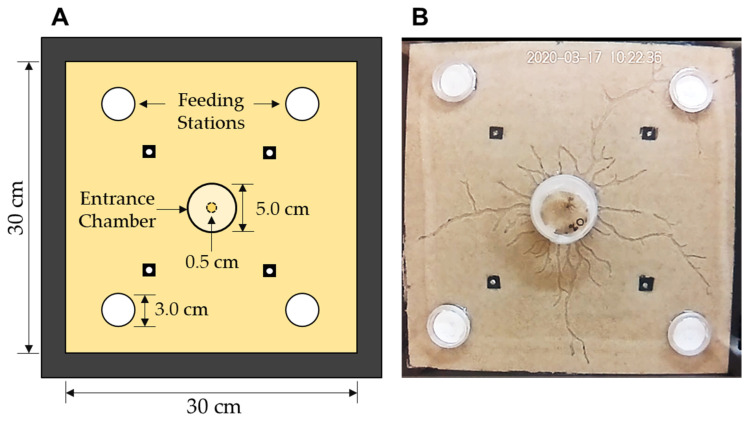
Experimental setup. (**A**) Foraging arena consisting of the sand substrate, four feeding stations, and a central entrance chamber; (**B**) Representative image of foraging tunnels at the end of 96 h, showing interception of one feeding station (indicated by arrow).

The foraging arena (Figure 1A) was adapted from a previous study [38]. It consisted of two clear Plexiglas sheets (35 × 35 × 0.6 cm^3^) divided by a border of black Plexiglas sheets (two pieces of 35 × 2.5 × 0.2 cm^3^, two pieces of 30 × 2.5 × 0.2 cm^3^), which created an open-area space of 30 × 30 × 0.2 cm^3^. Four Plexiglas spacers (1.0 × 1.0 × 0.2 cm^3^) were placed in the open area and glued to one of the Plexiglas sheets, each one 12 cm from one of the four corners. A hole was drilled through the Plexiglas sheets and each spacer, and a nut and bolt were used to secure the three layers, thus preventing the Plexiglas sheets from warping during the experiment. Five holes were drilled into the top layer of the Plexiglas sheet: one for an external entrance chamber and four for external food chambers (i.e., feeding stations). The holes drilled for the feeding stations were approximately 2.5 cm in diameter to ensure that, upon discovery, the termites encountered the filter paper. The entrance chamber (5.0 cm in diameter, 1.5 cm in height) was in the center, and each of the four food chambers (3.0 cm in diameter, 1.0 cm in height) were 6.0 cm from their closest respective corners. All chambers were fixed to the Plexiglas sheet with hot glue before the experiment to prevent the escape of termites or moisture. 

The arena was packed entirely with sand (1500 g, Quikrete Premium Play Sand, Atlanta, GA, USA) with 10% moisture (150 mL (dH_2_O): 1500 g (sand)). The entrance chamber was empty, and each of the feeding stations had two filter paper discs (3.0 cm in diameter, Whatman grade 1, Cytiva, Marlborough, MA, USA) that were pre-dried at 60 °C for 30 min and weighed. Each pair of filter paper received 30 μL of distilled water. Once in position, the Plexiglas sheets were bolted into place, and four 1-inch binder clips were clamped to each side of the arena to provide additional support. A layer of hot glue was applied along the perimeter of the arena to prevent water loss during the experiment. The lid of each food chamber was sealed with Parafilm (Parafilm M, Neenah, WI, USA) for the same reason. The entrance chamber was closed and sealed with a strip of Parafilm after the termites were introduced.

### 2.3. Foraging Behavior Assay

Four treatment groups consisting of 100 workers and either 0, 2, 10, or 30 soldiers were separated from their colony. We used a constant number of workers in each group to measure behaviors performed specifically by workers. A preliminary test of soldier-only foraging groups was conducted to ensure they could not tunnel or collect food on their own, and the results confirmed that none of the soldiers were able to tunnel out of their entrance chambers in the absence of workers. Prior to the assay, each foraging group was kept in an acclimation chamber (Petri dish 5.0 cm in diameter, 1.5 cm in height) with a piece of 3.0 cm filter paper wetted with 30 μL of distilled water. The acclimation chambers were stored for 24 h in their respective recording chambers at 25 °C in complete darkness. At the start of the experiment, the termites were gently moved from their acclimation dishes into their entrance chambers. Arenas were placed into their own recording chambers and kept at 25 ± 1 °C with constant, low light levels to reduce termite stress while effectively recording. Termites were recorded for 96 h using individual Raspberry Pi 3 Model B computers (Raspberry Pi, Cambridge, UK), each equipped with an Arducam OV5647 Lens Board Sensor for Raspberry Pi 3–4 with an Arducam M12 Interchangeable Lens (Arducam Technology, Kowloon, Hong Kong, China). 

Data on total tunnel length, number of tunnel intersections, and number of feeding stations intercepted were collected from clips of video recordings at 6, 24, 48, 72, and 96 h. Images from each time point were analyzed using ImageJ (version 1.53t, National Institutes of Health, Bethesda, MD, USA). The total tunnel length was determined by measuring the pixel length of all tunnels constructed by a foraging group at a given time. Pixel length in the approximate center of each tunnel was converted to centimeters using the 30 cm upper border of each arena as a reference measurement to determine the pixel-to-centimeter ratio for each image. Intersections were defined as any point where a tunnel branched into two or more directions for more than 0.5 cm and were counted at each time point in ImageJ. Feeding stations intercepted were recognized when at least one tunnel reached the food chamber. Filter paper discs that remained in the feeding chambers at the end of the assay were dried at 60 °C for 30 min and weighed again to measure food collection. Four colonies were used with four replicates from each colony for a total sample size of 16 for each treatment. 

### 2.4. Data Analysis

The effect of soldier proportions on tunnel length and number of intersections was assessed using linear mixed models (LMM) and negative binomial generalized linear mixed models (NB-GLMM), respectively, at each time point. The effects of soldier presence on workers’ food collection and the proportions of feeding stations intercepted were assessed using zero-inflated gamma (ziG-GLMM) and beta (ziB-GLMM) GLMMs. The numbers of feeding stations intercepted were divided by the total number of feeding stations prior to fitting the ziB-GLMMs. Each model included the colony of origin as a random intercept and slope, except in ziG-GLMM and ziB-GLMM, where it was included only as a random intercept. Model residuals, over-/under-dispersion, outliers, and zero-inflation were checked using a simulation-based approach via the package DAHRMa v0.4.5 [39]. Over-/under-dispersion, outliers, and zero-inflation were not observed, and the distribution of simulated residuals did not deviate significantly from the expected distribution of residuals for each model. Models were fitted via restricted maximum likelihood using the ‘lmer’ for LMMs and maximum likelihood with Laplace approximation using ‘glmer.nb’ for NB-GLMMs implemented in the R package lme4 v1-1.27.1 [40]. ziG-GLMMs and ziB-GLMMs were fitted using the function ‘glmmTMB’ with inverse and logit link functions, respectively, and checked for potential problems using the function “diagnose” implemented in the R package glmmTMB v1.1.3 [41]. The formula for zero-inflation included only the treatment variable. The overall significance of treatment coefficient estimates was assessed via Type II Wald Chi-square tests using the function “Anova” implemented in the R package car v3.1.0 [42]. All data analyses were performed on R v4.1.0 [43] and all figures were generated using JMP Pro 16 v16.2.0 (JMP Statistical Discovery, Cary, NC, USA). The original data were deposited in the Supplementary Materials (Table S1).

## 3. Results

**Figure 2:**
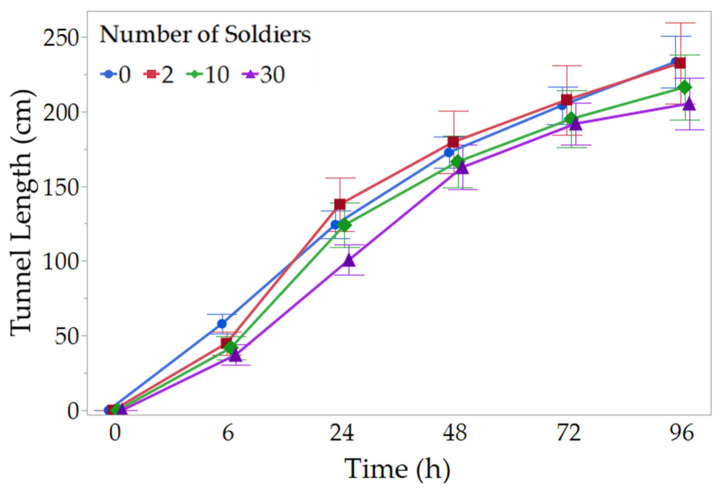
Changes in total tunnel length during exploratory foraging phase. Tunnels were constructed by groups of 100 foraging workers with different numbers of soldiers. Data shown are means ± SEM. No significant difference was detected among treatments at any time point (LMM, *p* > 0.05, n = 16).

**Figure 3:**
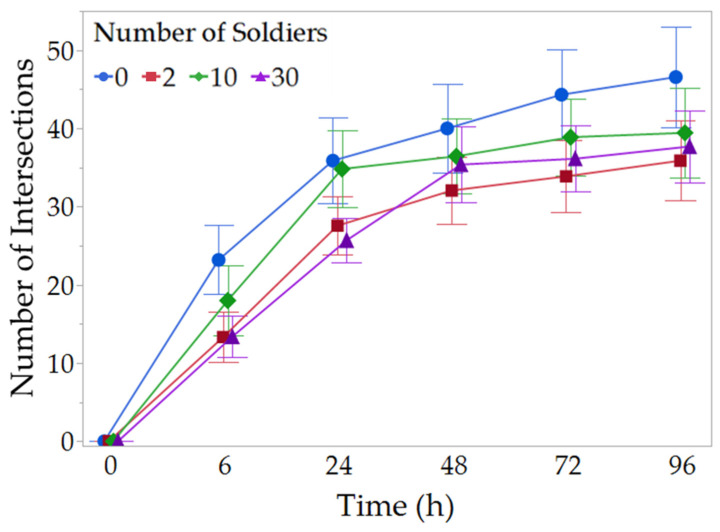
Changes in number of tunnel intersections during exploratory foraging phase. Tunnels were constructed by groups of 100 foraging workers with different numbers of soldiers. Data shown are means ± SEM. No significant difference was detected among treatments at any time point (NB-GLMM, *p* > 0.05, n = 16).

**Figure 4:**
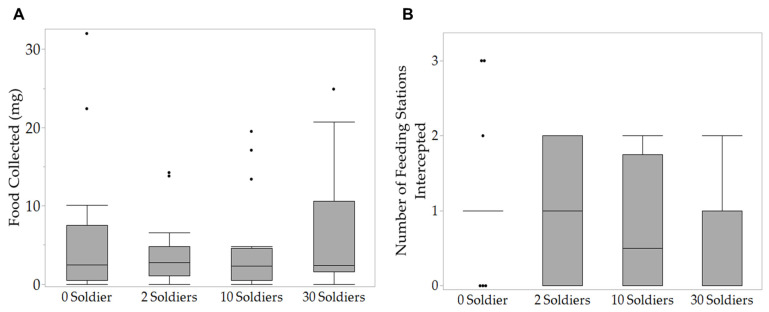
(**A**) Total food collection and (**B**) number of feeding stations intercepted in 96 h by groups of 100 foraging workers with different numbers of soldiers. Boxes are bounded by the 25th and 75th percentiles, bands are medians, whiskers represent minimum and maximum values, and dots outside of whiskers are outliers. No significant difference was detected among treatments (food collection: ziG-GLMM, *p* > 0.05; number of feeding stations intercepted: ziB-GLMM; *p* > 0.05, n = 16).

In all replications, workers initiated tunneling within 6 h. Termites performed the majority of their tunneling in the first 72 h, with most groups reaching the edge of their arenas between 24 and 48 h. The tunnel length was not significantly different between soldier treatment groups at any time point (LMM: 6 h: *X*^2^ = 2.07, *p* = 0.559; 24 h: *X*^2^ = 4.45, *p* = 0.217; 48 h: *X*^2^ = 0.83, *p* = 0.842; 72 h: *X*^2^ = 0.71, *p* = 0.870; 96 h: *X*^2^ = 1.56, *p* = 0.669; Figure 2). There were no significant differences in the number of tunnel intersections across treatments (NB-GLMM: 6 h: *X*^2^ = 4.18, *p* = 0.243; 24 h: *X*^2^ = 3.89, *p* = 0.274; 48 h: *X*^2^ = 1.34, *p* = 0.719; 72 h: *X*^2^ = 2.36, *p* = 0.501; 96 h: *X*^2^ = 2.05, *p* = 0.561; Figure 3). By the end of 96 h, there were no significant differences in the amount of food collected (ziG-GLMM: *X*^2^ = 1.70, *p* = 0.636; Figure 4A) or number of feeding stations intercepted (ziB-GLMM: *X*^2^ = 2.07, *p* = 0.557; Figure 4B) with various numbers of soldiers. 

## 4. Discussion

In this study, no significant differences were found in the exploratory foraging behaviors observed between workers in the absence or presence of soldiers in *C. formosanus*. The results add to the evidence that the influence of soldiers on foraging behavior is species-specific in subterranean termites. Soldier presence in *R. hageni* has been shown to influence the initiation of food exploration, and worker tunneling behavior was delayed by 31.8 h on average in the absence of soldiers [25]. In this study, *C. formosanus* workers initiated tunneling within 6 h regardless of soldier presence or proportion, and no significant difference in tunnel length was found in 6 h between treatment groups, suggesting little or no influence of soldiers on foraging initiation in *C. formosanus*. In *R. flavipes*, an effect of soldiers on tunnel initiation was not observed either; however, the presence of soldiers increased tunnel speed and branching [25]. Soldiers are not capable of excavating soil due to their morphologically modified mandibles for defense, and they affect tunneling by mediating behavioral plasticity of workers [25]. None of these effects were observed in *C. formosanus*, similar to previous findings in *R. virginicus* [25]. 

Subterranean termite colonies forage continuously, taking advantage of multiple food sources with foraging groups that move between the discovered food sources [44,45]. Our study showed that the *C. formosanus* soldier proportion had no significant effect on the number of feeding stations intercepted within 96 h. This implies that worker foraging exploration in this species may remain stable under dynamic changes in soldier composition. Furthermore, during the exploratory phase of foraging, the addition of different numbers of soldiers had no significant effect on food collection. Soldiers are incapable of feeding directly, and are thus fed by nestmate workers through trophallaxis [46]. An increase in total food collection is expected with the addition of soldiers when food is available because there are more termites consuming food overall. Our results may suggest that worker–soldier feeding does not occur until after exploration when a new food resource is colonized. Further investigations that consist of longer foraging periods and detailed behavioral observations are needed to validate when the provision of the soldier caste occurs in the colony. 

Different termite species exhibit a range of different foraging behaviors, even within subterranean termites. Workers of *R. flavipes* have been observed to tunnel faster and construct more branches than *R. hageni* and *R. virginicius* [25], and *C. formosanus* workers have been observed to construct longer tunnels but fewer branches than *C. gestroi* [47]. Additionally, *C. formosanus* colonies built shorter and wider primary tunnels compared to *R. flavipes* [48]. It is proposed that the differences in foraging strategy are partly dependent on resource abundance in the native range of the species, with longer and less branched tunnels associated with clumped and heterogeneous resources, and short and highly branched tunnels efficient for locating homogenously distributed resources [49]. The ecological drivers of different foraging strategies require further investigation in subterranean termite species. The presence of soldiers promoted the construction of tunnel branches in *R. flavipes* workers [25], likely reducing the chance of tunneling workers being attacked by predators and possibly increasing colony foraging efficiency. This impact was not observed in *C. formosanus*, suggesting that workers maintain foraging efficiency during food exploration regardless of variation in the number of soldiers present.

Under the conditions of this study, the results do not support the hypothesis that *C. formosanus* soldiers influence exploratory foraging behavior. Foraging workers, however, may be influenced by soldier presence in other social contexts. The behavioral influence of soldiers may be more obvious if foraging groups encounter predators or if their foraging galleries closely connect to the main nest where the reproductives or brood are present. Wells and Henderson noticed that groups of *C. formosanus* workers with low soldier proportions (~2.4%) explored new areas less frequently than groups with larger soldier proportions (~18%) in the presence of the red imported fire ant, *Solenopsis invicta* [50]. This indicates a potential role of soldiers in foraging exploration when predators are present. Worker foraging in *C. formosanus* was influenced by the presence of ant semiochemicals, the presence of which slowed tunneling, but that study did not explore whether soldier presence influenced this behavior [51]. Behavioral plasticity with respect to social context has also been studied in *R. speratus*, where soldiers and workers were more aggressive towards non-nestmate conspecifics when reproductive individuals were present rather than absent, and workers were less aggressive in the presence of nestmate soldiers [52]. The soldiers of *R. flavipes*, apart from influencing worker tunneling speeds [10], also alleviated worker stress in the presence of a competing termite species, *R. virginicius*, even without direct contact with the competitors [24]. 

The soldier proportion of *C. formosanus* (~10%) is relatively high compared to subterranean termites of other genera, such as *Reticulitermes* (~2%) and *Hetermotermes* (2–5%) [7], while it is similar to other species of *Coptotermes*, including *C. heimi* (~33%), *C. vastator* (~9%), and *C. intermedius* (9–17%) [7,30]. *C. formosanus* is native to southeast Asia [53], a subtropical region with a wide variety of predators and competitors. *Reticulitermes* species, however, are distributed primarily in temperate regions [11]. A review by Tuma, Eggleton, and Fayle on ant–termite interactions [22] updated the previously published genus richness maps of termites [54] and ants [55], and showed that both ant and termite diversity were higher in the native range of *C. formosanus* than the native range of *Reticulitermes* species. While the difference in soldier proportions between *C. formosanus* and *Reticulitermes* colonies are likely due to multiple factors, the greater predatory and competitive pressures *C. formosanus* faced in their native range may have been a significant driver. Wells and Henderson supported this hypothesis by showing that *C. formosanus* fought off ant predators more successfully than *Reticulitermes* sp. at their typical range of soldier proportions (18.3% and 2.3%, respectively), but not when their soldier proportion was reduced from 18.3% to 2.3% [50]. 

Thus far, there is still limited understanding of the potential mechanisms behind the roles that termite soldiers play within the colony. Soldier–worker interaction is widespread in termites, but the influence on colony-level behavior and the regulatory cues have been determined in few species [2]. In *R. speratus* (Kolbe), soldiers can elicit arrestant behaviors from workers using soldier-specific volatile (-)-*ß*-elemene, possibly to encourage worker–soldier feeding or grooming [56]. Soldier presence in *C. formosanus* can also affect juvenile hormone titers, allowing for the self-regulation of worker–soldier differentiation [57]. The cuticular hydrocarbon profiles of *C. formosanus* soldiers and workers were quantitatively different, suggesting a potential mechanism of caste recognition in this species [58]. However, soldier pheromones and their function beyond colony defense await further exploration in *C. formosanus* and other related species of economic significance, such as *C. gestroi*. Further studies focusing on the behavioral influences of soldiers on workers or other castes will provide a better understanding of the complex and cryptic nature of social behavior in subterranean termites. 

## Data Availability

The data presented in this study are available in the Supplementary Materials.

## Supplementary Materials

The following supporting information can be downloaded at: https://www.mdpi.com/article/10.3390/insects14020198/s1; Table S1: original data.
